# TLR3 engagement induces IRF-3-dependent apoptosis in androgen-sensitive prostate cancer cells and inhibits tumour growth *in vivo*

**DOI:** 10.1111/jcmm.12379

**Published:** 2014-12-02

**Authors:** Guido Gambara, Marianna Desideri, Antonella Stoppacciaro, Fabrizio Padula, Paola De Cesaris, Donatella Starace, Andrea Tubaro, Donatella del Bufalo, Antonio Filippini, Elio Ziparo, Anna Riccioli

**Affiliations:** a Istituto Pasteur-Fondazione Cenci Bolognetti, Department of Anatomy, Histology, Forensic Medicine and Orthopedics, Section of Histology and Medical Embryology, Sapienza University of RomeRome, Italy; b Experimental Chemotherapy Laboratory, Regina Elena National Cancer InstituteRome, Italy; b Department of Clinical and Experimental Medicine, Sapienza University of Rome, Sant'Andrea HospitalRome, Italy; d Department of Biotechnological and Applied Clinical Sciences, University of L'AquilaL'Aquila, Italy

**Keywords:** toll-like receptor, poly I:C, IRF-3, apoptosis, prostate cancer

## Abstract

Toll-like receptors (TLRs) are a family of highly conserved transmembrane proteins expressed in epithelial and immune cells that recognize pathogen associated molecular patterns. Besides their role in immune response against infections, numerous studies have shown an important role of different TLRs in cancer, indicating these receptors as potential targets for cancer therapy. We previously demonstrated that the activation of TLR3 by the synthetic double-stranded RNA analogue poly I:C induces apoptosis of androgen-sensitive prostate cancer (PCa) LNCaP cells and, much less efficiently, of the more aggressive PC3 cell line. Therefore, in this study we selected LNCaP cells to investigate the mechanism of TLR3-mediated apoptosis and the *in vivo* efficacy of poly I:C-based therapy. We show that interferon regulatory factor-3 (IRF-3) signalling plays an essential role in TLR3-mediated apoptosis in LNCaP cells through the activation of the intrinsic and extrinsic apoptotic pathways. Interestingly, hardly any apoptosis was induced by poly I:C in normal prostate epithelial cells RWPE-1. We also demonstrate for the first time the direct anticancer effect of poly I:C as a single therapeutic agent in a well-established human androgen-sensitive PCa xenograft model, by showing that tumour growth is highly impaired in poly I:C-treated immunodeficient mice. Immunohistochemical analysis of PCa xenografts highlights the antitumour role of poly I:C *in vivo* both on cancer cells and, indirectly, on endothelial cells. Notably, we show the presence of TLR3 and IRF-3 in both human normal and PCa clinical samples, potentially envisaging poly I:C-based therapy for PCa.

## Introduction

Prostate cancer (PCa) is the second most frequent diagnosed cancer among males worldwide and it is the sixth leading cause of cancer-related deaths 1. Standard therapy, consisting in surgical excision of the prostate or radiation, initially leads to regression of the disease, which however is often transient and no cure is known for late stage PCa. Consequently, many efforts are being made to identify novel molecular targets for the prevention and treatment of this disease.

Toll-like receptors (TLRs) are a family of transmembrane proteins that recognize pathogen associated molecular patterns, molecules highly conserved in bacteria, viruses, fungi and parasites 2,3. TLRs are the key sensors of the innate immunity and are critically involved in priming the adaptive immune response necessary for killing invading pathogens 4–6. The first event of TLR signal transduction is the microbial ligand-induced TLR dimerization generating a TIR-TIR (Toll/Il-1 Receptor) domain interface able to recruit adaptor proteins 7. TLR3 signals specifically through TIR domain-containing adaptor inducing interferon-β (TRIF), while the other TLRs recruit the myeloid differentiation factor 88 (MyD88) with the exception of TLR4 that signals through both TRIF and MyD88 3. TLR ligands activate mitogen-activated protein kinases (MAPKs) 8, NF-κB, interferon regulatory factors (IRFs) and other transcription factors regulating proliferation and most of inflammation-linked genes 3.

Besides the canonical antimicrobial function of TLRs, it has been demonstrated that these receptors are expressed in cancer epithelial cells and involved in the control of tumour growth 9. Interestingly, although conflicting reports have been published concerning the pro- or antitumoural role of several TLRs, most literature data agree on an antitumour role for TLR3 10,11. Different hypothetic functioning modes for TLR3-dependent anticancer mechanism have been proposed 12: (*i*) An immune-mediated tumour growth suppression 13, (*ii*) a direct apoptotic effect on TLR3 expressing cancer cells 14–17, (*iii*) an inhibition of tumour growth through both immune-mediated anticancer mechanisms and cancer cell apoptosis. Our group has previously demonstrated that activation of TLR3 in PCa cell lines induces the secretion of cytokines and chemokines that can recruit and activate immune cells in the tumour site consequently promoting their anticancer activity 18. We have also shown that the synthetic TLR3 agonist poly I:C inhibits the proliferation and induced apoptosis in LNCaP and PC3 cells, with much higher efficiency in the former than in the latter more aggressive line, depending on differential degree of up-regulation of the powerful tumour shield, hypoxia inducible factor-1 19. In detail, poly I:C elicits inhibition of proliferation associated with a marked induction of TLR3-mediated apoptosis in LNCaP cells through a PKC-α dependent mechanism upstream to MAPK phosphorylation 15. Nevertheless, several steps along this pro-apoptotic signalling pathway still need to be clarified, in this work we investigated other molecules involved in poly I:C-induced apoptosis in LNCaP cells and the *in vivo* efficacy of poly I:C-based cancer therapy.

Toll-like receptor 3 is engaged by double-stranded RNA (dsRNA) that represents either genomic or life cycle intermediates of many viruses. In infected cells TLR3 ligands trigger the activation of signalling pathways involved in antiviral responses, leading to IRF-3-induced IFN production and NF-κB-induced cytokines and chemokines secretion 20. Focusing on IRF-3 activation, it has been shown that dsRNA or poly I:C bind TLR3 promoting the interaction of TRIF with TBK-1 and the resulting migration of phosphorylated IRF-3 into the nucleus 21,22. Then nuclear IRF-3 binds to interferon-stimulated response elements promoting the transcription of type I IFNs and other genes involved in immune response 23. Besides its key role in antiviral immunity, IRF-3-mediated apoptosis in response to dsRNA was observed in melanoma and fibrosarcoma cells 24. Our present data show that IRF-3 is a crucial player in poly I:C induced apoptosis in LNCaP cells.

Moreover, we hypothesize a key role of direct apoptotic effect in the antitumoural function of poly I:C *in vivo* regardless of the well-known effectiveness of this compound as adjuvant in anticancer immunotherapy 25. In the present work, we verified this hypothesis in PCa showing that subcutaneous growth of human LNCaP cells in immunodeficient NOD scid gamma (NSG) mice was severely impaired by treatment with poly I:C, thus demonstrating the direct anticancer effect of the TLR3 agonist as a single therapeutic agent in human PCa *in vivo*. Moreover, to evaluate the presence of TLR3-IRF-3 axis in the human prostate we performed an immunohistochemistry analysis on normal human prostate epithelium and PCa tissues. Expression of TLR3 and IRF-3 was found in human prostate tissues both normal and tumoural demonstrating that TLR3 signalling components are not lost in PCa.

## Materials and methods

### Cell lines

Non-neoplastic adult human prostatic epithelial cells were immortalized with human papillomavirus 18 leading to establishment of the RWPE-1 cell line 26.

RWPE-1 and LNCaP cell lines were obtained from American Type Tissue Culture Collection (Rockville, MD, USA). LNCaP C4-2B were kindly provided by Dr. Lien Spans (University of Leuven, Leuven, Belgium).

RWPE-1 were maintained in keratinocyte SFM (serum-free medium) supplemented with epidermal growth factor (5 ng/ml), bovine pituitary extract (0.05 mg/ml; Gibco, Paisley, UK) and antibiotics (penicillin 10 U/ml and streptomycin 10 μg/ml). LNCaP were maintained in RPMI medium supplemented with 2 mM L-glutamine, 100 IU/ml penicillin-streptomycin and 10% foetal calf serum (FCS; Sigma-Aldrich, St Louis, MO, USA). LNCaP cells at low passages (23–33) were used to perform all the experiments. LNCaP C4-2B were maintained in DMEM-F12 medium supplemented with 2 mM L-glutamine, 100 IU/ml penicillin-streptomycin and 10% FCS. For signal transduction experiments, cells were serum starved for 24 hrs and then stimulated with poly I:C in FCS-free medium; otherwise, cells were treated in medium containing 3% FCS.

### Reagents

Poly I:C was from Invivogen (San Diego, CA, USA). Z-IETD-FMK, Z-LEHD-FMK and BX-795 were purchased from Merck-Millipore (Billerca, MA, USA). Bicalutamide was purchased from Tocris Bioscience (Bristol, UK). Unless otherwise specified reagents were purchased from Sigma-Aldrich.

### Immunofluorescence

Imaging of fluorescent markers was performed on cells grown on glass coverslips. The nuclear translocation of IRF-3 was analysed in cells treated for 4 hrs with 25 μg/ml poly I:C, then fixed in 4% paraformaldehyde, permeabilized with PBS/0.1% Triton X-100 and immunostained with primary anti-IRF-3 polyclonal antibody (BD Biosciences, Milano, Italy) followed by an Alexa Fluor 555-conjugated secondary anti-rabbit antibody (Molecular Probes, Invitrogen, Eugene, Oregon, USA).

### Apoptosis assays

Propidium iodide (PI) staining: cells were detached with trypsin, washed with cold PBS-5% FCS and then fixed in 70% ethanol for 24 hrs, at 4°C. After washing with PBS, cells were incubated with 1 μg/ml PI for 3 hrs at 25°C before FACS analysis by Coulter Epics XL flow cytometer (Beckman Coulter, Fullerton, CA, USA). Data were analysed by using FCS Express by De Novo Software (Los Angeles, CA, USA). Cells were considered apoptotic when their DNA content was <2*N* after excluding debris.

AnnexinV staining: Cells were detached with trypsin, washed with PBS–5% FCS and then placed in binding buffer containing 0.14 M NaCl, 2.5 mM CaCl_2_ and 0.01 M *N*-2-hydroxyethylpiperazine-*N*′-2-ethanesulfonic acid (pH 7.4) to which Propidium Iodine and annexin V-FITC (Immunological Sciences, Rome, Italy) were added prior to FACS analysis following the manufacturer's instructions. Annexin V-FITC positive and P.I. negative cells were considered apoptotic.

### siRNA silencing

Small interfering RNA duplex oligonucleotides complementary to the 3′UTR of human IRF-3 (5′-GGA CCA AGA GGC UCG UGA UGG UCA A-3′) and Noxa (5′-GCA UUG UAA UUG AGA GGA AUG UGA A-3′) were synthesized and purchased from Integrated DNA Technologies (Coralville, IA, USA). Transfection of 10 nM siRNA in LNCaP cells was carried out with Oligofectamine Transfection Reagent, according to the manufacturer's instructions. Complete fresh medium was added 5 hrs after transfection and cells were further incubated for 42–72 hrs prior to proceeding with other treatments. Non-targeting control served as experimental control.

### Caspase activity assay

Caspase activity was evaluated using the caspase assay kit (BioVision, Milpitas, CA, USA) as recommended by the manufacturer. LNCaP cell lysates were evaluated for their ability to cleave substrates specific for recognition by active caspase 8 (IETD-*p*NA) and caspase 9 (LEHD-*p*NA). After 2–4 hrs at 37°C, samples were read with a plate reader (das, Italy) at 405 nm.

### Western blotting

Cell lysates were prepared in cell lysis buffer (Cell Signaling, Danvers, MA, USA). Protein concentration was determined by the micro bicinchoninic acid method (Pierce, Rockford, IL, USA).

Equal amounts of proteins (25–40 μg) were subjected to SDS-PAGE and transferred onto nitrocellulose membrane. Then membranes were saturated with 5% non-fat dry milk in Tris-buffered saline with 0.1% Tween-20 and incubated overnight with primary antibody at 4°C and subsequently with horseradish peroxidase-conjugated secondary antibody for 1 hr at room temperature. Membranes were washed with Tris-buffered saline with 0.1% Tween-20 after each antibody incubation and developed by using the chemiluminescence system (ECL Advance; Amersham Bioscience, Piscataway, NJ, USA). Source of primary antibodies: anti-phospho-IRF-3, anti-TLR3 and anti-Cleaved Caspase 3 from Cell Signaling (Beverly, MA, USA), anti-NOXA from Enzo Life Science (Lausen, Switzerland) and β-actin antibodies from Sigma-Aldrich. Horseradish peroxidase-conjugated goat antimouse or anti-rabbit Secondary antibodies were from Bio-Rad (Hercules, CA, USA).

### *In vivo* model

Six to 10-week-old male NOD scid gamma (NOD.Cg-Prkdc^scid^Il2rg^tm1Wjl^/SzJ) mice were purchased from Charles River Laboratories (Calco, LC, Italy) and housed in groups of four in isolated ventilated cages; food and water were provided *ad libitum*. All animal procedures were performed according to the protocol approved by the Istituto Superiore di Sanità Animal Care Committee (DM No. 228/2009). 4 × 10^6^ LNCaP cells were resuspended in 100 μl PBS and mixed (1:1, vol/vol) with growth factor reduced matrigel (BD Biosciences, San Jose, CA, USA). The cells suspension was subcutaneously injected into the flank of mice. Tumours were grown to ≅50–100 mm^3^ before beginning treatment with poly I:C or vehicle (saline solution). Intraperitoneal or intratumoural injection of 16 mg/kg poly I:C was performed 3 times weekly for 3–4 weeks. Tumour volume based on calliper measurements were calculated by the modified ellipsoidal formula (Tumour volume = ½ × (length × width^2^)).

### Immunohistochemistry

Xenografts were harvested for histological analysis, embedded in OCT frozen in liquid nitrogen and preserved at −80°C until sectioning.

After personal patient consent and Sant'Andrea Hospital Ethical Comity approval, 30 human PCas blocks, 12 from radical prostatectomy and 18 from diagnostic biopsies, from the histoteque of Sant'Andrea Hospital Pathology Section of Clinical and Molecular Department of ‘Sapienza’ University of Rome, were chosen to have 10 cases with Gleason score (as modified by WHO 2011) ≤6 (G1), 10 cases Gleason score = 7 (G2), 10 cases Gleason score ≥8 (G3).

5 μm mouse tumours frozen sections, fixed with 100% acetone and 5 μm human cancer paraffin sections deparaffined and antigen retrieved by using a pH9 Dako Retriever solution, were immunostained by a DAKO Autostainer, with EnVision™ FLEX+ revelation system (Dako Colorado Inc, Fort Collins, Colorado, USA). Primary antibody Company, Clones, Isotype and working dilutions are reported in Table S1.

Human PSA, Ki67 and AMACR were diluted and used as routinely done during prostate adenocarcinoma diagnostic workout. For antibodies against CXCR4, VEGF, IRF3, CD34, TLR3, Cl. Caspase 3, working dilutions were established by using different frozen or/and formalin fixed, paraffin embedded control tissues such as human reactive lymph nodes for CXCR4, IRF3, TLR3 and active caspase-3, Placenta for VEGFA and finally mouse lung for CD34. Negative controls were done by using the respective control isotype antibodies (Table S1). Controls for the secondary antibodies were done withdrawing the primary antibodies. Tissues reactivity was finally compared with the THE HUMAN PROTEIN ATLAS (http://www.proteinatlas.org/). Mouse derived tumour results are reported as a mean + SD of the percentage of positive cells.

### Statistical analysis

Differences between groups were analysed with a two-sided paired or unpaired Student's *t*-test by use of GraphPad Prism 5.00 (GraphPadSoftware, San Diego, CA, USA). Results were considered to be statistically significant if *P* < 0.05.

## Results

### Crucial role of IRF-3 in TLR3-mediated apoptosis in LNCaP cells

**Fig 1:**
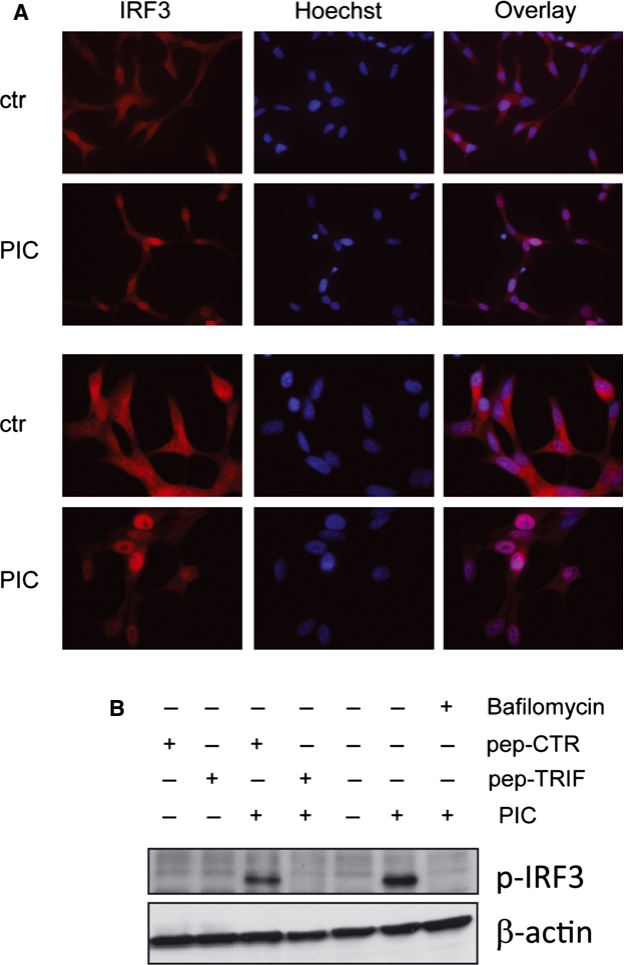
Poly I:C induces IRF-3 activation in LNCaP cells. (A) LNCap cells were stimulated with 25 μg/ml poly I:C (PIC) for 4 hrs and stained with IRF-3 antibody (Red) and Hoechst (blue). Nuclear translocation of IRF-3 in PIC treated cell is shown. Original low magnification 400X, high magnification 630X. (B) WB analysis showing the inhibition of PIC-induced IRF-3 phosphorylation (Ser-396) by pre-treatment of LNCaP cells with 25 μM TRIF inhibiting peptide (pep-TRIF) or control peptide (pep-CTR) or 100 nM Bafilomycin A1. β-actin was used as control for equal amount of proteins loaded. Similar results were obtained in three independent experiments.

The transcription factor IRF-3 plays a crucial role in dsRNA-induced apoptosis of infected cells 27. Therefore, we wondered whether IRF-3 is also involved in TLR3-mediated apoptosis in the poly I:C-sensitive PCa cells LNCaP. Firstly, we verified the activation of IRF-3 in LNCaP stimulated with 25 μg/ml poly I:C for 4 hrs by evaluating the ability of IRF-3 to migrate to the nuclei by immunofluorescence analysis. IRF-3 was observed to co-localize with DNA stained with Hoechst after poly I:C stimulation (Fig.1A). IRF-3 activation was also confirmed by western blot showing IRF-3 phosphorylation in poly I:C-treated cells (Fig.1B). Subsequently, we verified whether poly I:C-induced IRF-3 activation is dependent on TLR3 or on different cytosolic receptors able to sense dsRNA and priming innate immune response. To this aim, we pre-treated cells with an inhibitor of endosome acidification (Bafilomycin A1), known to impair TLR3 signalling. As expected, Bafilomycin A1 completely inhibited poly I:C-induced IRF-3 phosphorylation (Fig.1B). To further confirm the exclusive role of TLR3 upstream IRF-3 phosphorylation, we inhibited TRIF-1, a molecule which is found exclusively downstream of TLR3, by pre-incubating cells with the membrane permeable TRIF-1 inhibiting peptide (pepinh-TRIF) or a control peptide (pepinh-CTR). Western blot analysis showed that, treatment with 25 μM pepinh-TRIF completely blocks poly I:C-induced phosphorylation of IRF-3 indicating that TLR3 triggers IRF-3 activation in LNCaP cells (Fig.1B).

**Fig 2:**
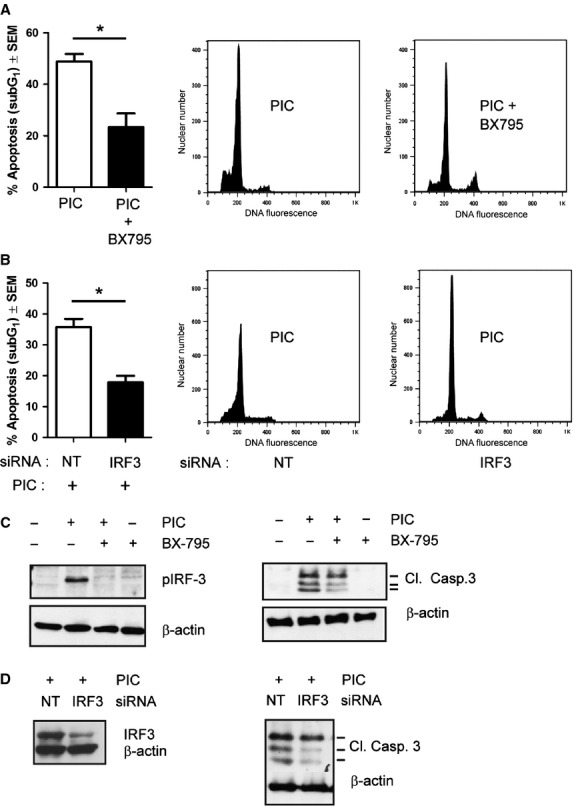
IRF-3 regulates Poly I:C-induced apoptosis in LNCaP cells. (A) 1 μM TBK-1 inhibitor BX-795 significantly reduced apoptosis in LNCaP cells stimulated with 25 μg/ml poly I:C (PIC) for 24 hrs. (B) IRF-3 silencing impaired apoptosis in LNCaP cells stimulated with 25 μg/ml PIC for 24 hrs (NT = non-targeting). (A and B) Bar chart represents % of SubG_1_ cells (mean ± SEM) of three independent experiments in duplicate (left panel) *P < 0.006. Representative plots of cell cycle analysis evaluated by flow cytometry (PI staining) are shown (Right panel). The range of apoptosis of Ctrl is 0–1%. (C and D) LNCaP cells were treated with 25 μg/ml PIC for 24 hrs in the presence of BX-795 (C) or following IRF-3 siRNA (D). Whole cell lysates were analysed by western blot with anti-IRF-3, anti-phospho-IRF-3 and anti-Cleaved Caspase 3 (Cl. Casp. 3) antibodies. β-actin was used as control for equal amount of proteins loaded. Similar results were obtained in three independent experiments.

To investigate the potential involvement of IRF-3 in TLR3-mediated apoptosis we used BX-795, an inhibitor of TBK-1 (the kinase responsible for IRF-3 activation), and evaluated its ability to inhibit the apoptotic effect of poly I:C. PI staining showed that BX-795 pre-treatment significantly inhibits poly I:C-induced apoptosis. Indeed, flow cytometry analysis showed a reduction of sub-G1 peak from 48.9 ± 2.9% in poly I:C-treated cells to 23.4 ± 5.2% in cells pre-treated with BX-795 and stimulated with poly I:C (Fig.2A). To confirm the involvement of IRF-3 in poly I:C-induced apoptosis, we performed specific knock-down of IRF-3 by small interfering RNA in LNCaP cells and we observed that IRF-3 silencing strongly inhibits apoptosis in cells stimulated with poly I:C compared to cells pre-treated with non-targeting siRNA (sub-G_1_ apoptotic cell population = 17.84% *versus* 35.74%; Fig.2B). As a control, we verified that BX-795 treatment inhibited poly I:C-induced IRF-3 phosphorylation (Fig.2C) and that targeting of IRF-3 by means of siRNA sequence massively reduced IRF-3 protein expression, as shown in Figure2D.

Moreover, we assayed cleaved caspase-3 levels in LNCaP cells in which the activation or the expression of IRF-3 was pharmacologically or genetically reduced. Western blot analysis showed that both IRF-3 phosphorylation inhibitor BX-795 and IRF-3 silencing strongly inhibit caspase-3 cleavage (Fig.2C and D), confirming the role of IRF-3 in TLR3 mediated apoptosis in LNCaP cells.

### Poly I:C triggers IRF-3-dependent extrinsic and intrinsic apoptotic pathways

**Fig 3:**
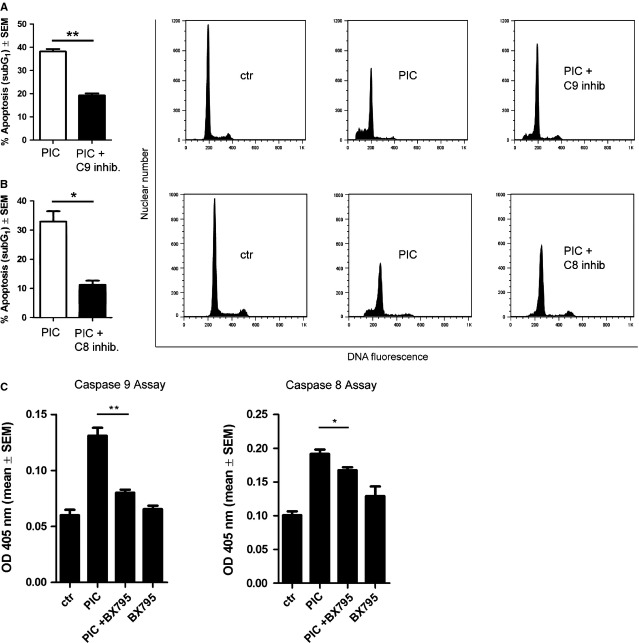
IRF-3 regulates PIC-induced caspase 8 and 9 activity. (A and B) Bar charts (left panel) represent % of SubG_1_ cells (mean ± SEM) stimulated with poly I:C (PIC) for 24 hrs in the presence or absence of caspase 9 (A) or caspase 8 (B) inhibitors. Results are from three independent experiments in duplicate. Representative plots of cell cycle analysis evaluated by flow cytometry are shown (A and B, right panel); *P < 0.005; **P < 0.0003. The range of apoptosis of Ctrl is 0–1%. C, BX-795 inhibits caspase 8 and 9 activity. Bar chart represents OD 405 nm (mean ± SEM) of three independent experiments; *P < 0.04, **P < 0.003.

To determine whether in LNCaP cells poly I:C induces apoptosis and triggers a caspase cascade through extrinsic and/or intrinsic pathways, we evaluated the effect of the specific caspase 9 inhibitor Z-LEHD-FMK or of the caspase 8 inhibitor Z-IETD-FMK in poly I:C-induced apoptosis. As shown in Figure3A and B, Z-LEHD-FMK-treated poly I:C-stimulated LNCaP cells exhibited an apoptotic rate reduction of 50%, whereas caspase 8 inhibitor appears to be more effective in reducing poly I:C-induced apoptosis (67%). In detail, sub-G_1_ apoptotic cell population in poly I:C plus Z-IETD-FMK-treated cells is 11.2% ± 1.4% compared to 32.9% ± 3.5% in cells treated with poly I:C alone. Therefore, to analyse the role of IRF-3 in caspase activation we investigated the effect of the TBK-1 inhibitor BX-795 on the activity of caspase 9 and 8 in LNCaP cells. As expected, 25 μg/ml poly I:C increased activity both of caspase 9 and 8 and 1 μM BX-795 treatment massively reduced caspase 9 activity (O.D.: 0.131 *versus* 0.08), while slightly reducing poly I:C-induced caspase 8 activity (O.D.: 0.192 *versus* 0.167; Fig.3C). These data suggest that IRF-3 activation represents a critical step in caspase-8 and 9-mediated apoptotic pathway, even though at different extent.

### Noxa silencing increases TLR3-mediated apoptosis

**Fig 4:**
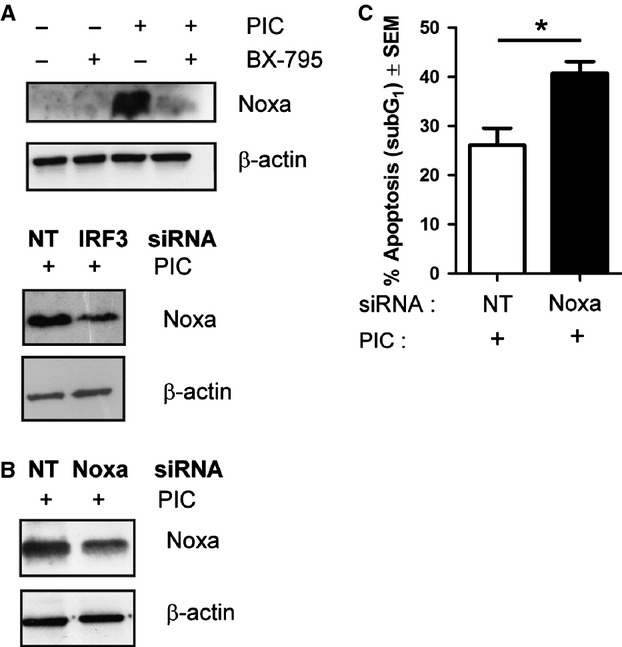
Noxa silencing increases TLR3-mediated apoptosis in LNCaP cells. (A) western blot analysis showing the inhibitory effects of BX-795 (1 μM) and IRF-3 silencing on poly I:C-induced expression of Noxa. (B) Western blot analysis after Noxa silencing in cells stimulated with poly I:C for 4 hrs. β-actin was used as control for equal amount of proteins loaded. (C) Evaluation of apoptosis by means of PI staining. Bar chart represents % of SubG_1_ cells (mean ± SEM) stimulated with PIC in presence of non-targeting (NT) or Noxa siRNA (*P < 0.03). The range of apoptosis of Ctrl is 0–3%.

To assess the mechanism by which IRF-3 regulates TLR3-mediated apoptosis, we examined the modulation of pro-apoptotic proteins in LNCaP cells in which the activation or the expression of IRF-3 was pharmacologically or genetically altered. Despite the role of IRF-3 in regulating type I IFN transcription, it has been shown that this transcription factor also regulates the expression of pro-apoptotic proteins involved in the intrinsic apoptotic pathway, such as Noxa 24. Consistent with this evidence, we observed that Noxa is overexpressed in poly I:C-treated LNCaP cells and that BX-795 treatment inhibits Noxa induction (Fig.4A), indicating Noxa as a potential pro-apoptotic effector of TLR3 signal transduction *via* IRF-3. To better elucidate the role of IRF-3 in this process silencing experiments were performed. IRF-3 down-regulation inhibited the overexpression of Noxa induced by poly I:C, confirming the essential role of IRF-3 in the control of Noxa expression (Fig.4A). Surprisingly, silencing of Noxa (Fig4B) increased poly I:C-induced apoptosis in LNCaP cells, as shown in Figure4C.

### LNCaP cells are more sensitive to poly I:C-induced apoptosis than normal prostate epithelial cells

**Fig 5:**
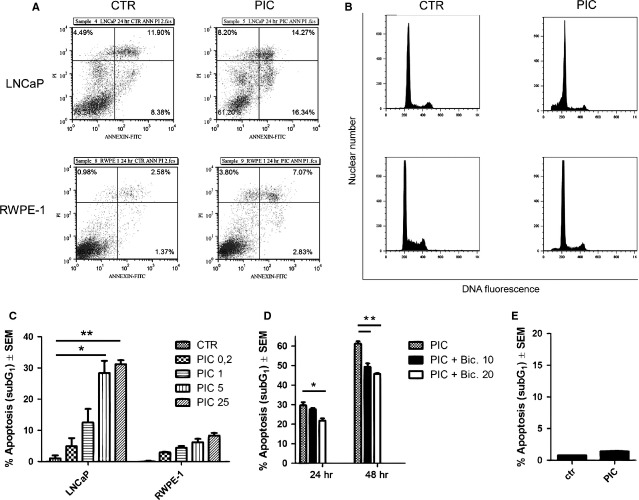
Poly I:C induces selectively apoptosis in androgen-sensitive prostate cancer cells. (A–C) Flow cytometric analysis of apoptosis. LNCaP and RWPE-1 cells were stimulated with 25 μg/ml poly I:C (PIC) for 24 hrs and the percentages of apoptotic cells were determined by flow cytometry by using Annexin-FITC/Propidium Iodide dual staining (PI; A) and SubG_1_ peak analysis after PI staining (B). (C) The histogram shows the apoptotic effect of increasing doses of PIC in LNCaP and RWPE-1 cells evaluated by PI staining. PIC concentrations are expressed as μg/ml. Data represent mean ± SEM of three independent experiments. *P < 0.03, **P < 0.0005. (D) SubG_1_ peak analysis of LNCaP cells pre-treated 1 hr with 10 or 20 μM bicalutamide (Bic.) and then stimulated with 25 μg/ml PIC, as indicated. *P < 0.02, **P < 0.0065. (E) Histogram showing the effect of 25 μg/ml PIC in LNCaP C4-2B cells.

To determine the selectivity of poly I:C, we evaluated its apoptotic effect in normal prostate epithelial cells RWPE-1. Firstly, we showed that TLR3 was expressed in RWPE-1 and LNCaP cells and up-regulated in the two cell lines stimulated with poly I:C for 24 hrs (Fig. S1). Subsequently, the ability of poly I:C to induce apoptosis was investigated by means of flow cytometry. Annexin V-FITC/PI staining showed that LNCaP cells were more sensitive to poly I:C-induced apoptosis than RWPE-1 (annexin V-FITC^+^/PI^−^: 16.34% *versus* 2.83%; Fig.5A). These data were confirmed by PI staining assay showing an increase of sub-G_1_ peak (apoptotic cells) up to ≅30% in LNCaP cells after 24 hrs treatment with 25 μg/ml poly I:C (*P* < 0.002; Fig.5B). Poly I:C-induced apoptotic rate rose in a remarkable dose-dependent manner in LNCaP cells, whereas only slightly increased (less than 10%) in RWPE-1 cells (Fig.5C).

### Androgen sensitivity is crucial for poly I:C-induced apoptosis in LNCaP cells

Since we have previously demonstrated that poly I:C induces a stronger apoptosis in androgen-sensitive LNCaP cells compared with androgen-insensitive AR negative PC3 and DU-145 cell lines 10,15, we treated LNCaP cells with androgen receptor (AR) inhibitor bicalutamide to evaluate the potential contribution of androgen sensitivity to poly I:C-induced apoptosis in LNCaP cells. Flow cytometry analysis showed that when poly I:C-stimulated cells were also pre-treated with 10 or 20 μM bicalutamide, the sub-G_1_ peak was significantly reduced as shown in Figure5D. Moreover, the metastatic, androgen-insensitive and AR positive LNCaP subclone C4-2B 28 resulted completely unresponsive to poly I:C-induced apoptosis, as shown in Figure5E.

### *In vivo* TLR3-mediated inhibition of tumour growth

**Fig 6:**
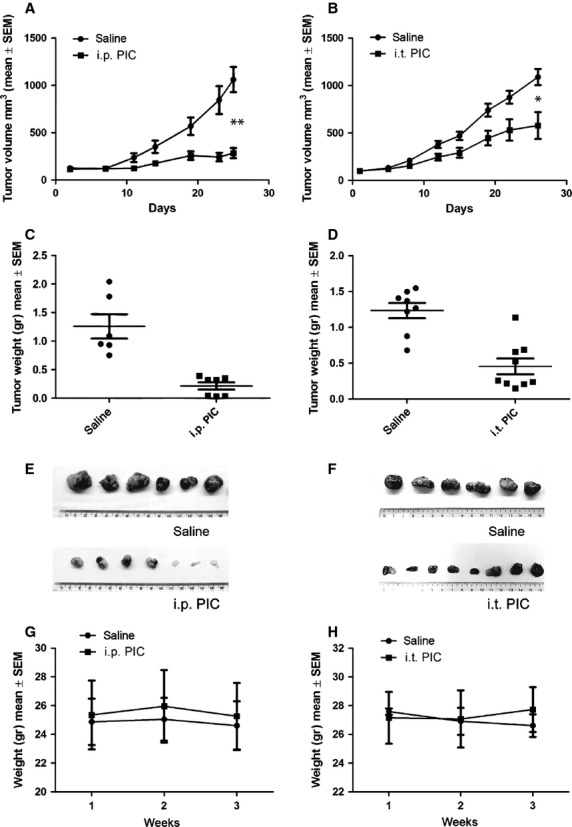
Poly I:C inhibits the growth of LNCaP xenografts. LNCaP cells were subcutaneously inoculated in NSG mice and treated as indicated in Material and Methods. Analysis of tumour volume (A and B) and tumour weight of (C and D) mice injected intraperitoneal (i.p.) (A and C) or intratumoural (i.t.) (B and D) with poly I:C or saline and representative pictures of tumours treated as indicated (E and F). Graphs show the bodyweight of mice bearing tumour xenografts treated with i.p. (G) or i.t. (H) poly I:C or saline solution as indicated. Graph of a representative experiment (mean ± SEM). Comparable results were obtained in three independent experiment for i.p. treatment and two independent experiments for intratumoural treatment. *P < 0.006; **P < 0.002. Each group consists of minimum 6 mice.

To investigate the direct effect of poly I:C on tumour growth *in vivo*, we inoculated LNCaP cells in NSG mice (lacking B, T and NK cells). Our results show that tumour growth was severely impaired by both intraperitoneal (Fig.6A, C and E) and intratumoural (Fig.6B, D and F) poly I:C administration, demonstrating for the first time the direct non-immune-mediated anticancer effect of the TLR3 agonist as a single therapeutic agent in human PCa cells *in vivo*.

Intraperitoneal or intratumoural injection of 16 mg/kg poly I:C 3 times weekly for 3–4 weeks did not produce any adverse health effects in mice as monitored by diet consumption, bodyweight loss (Fig.6G and H) and postural and behavioural changes.

**Fig 7:**
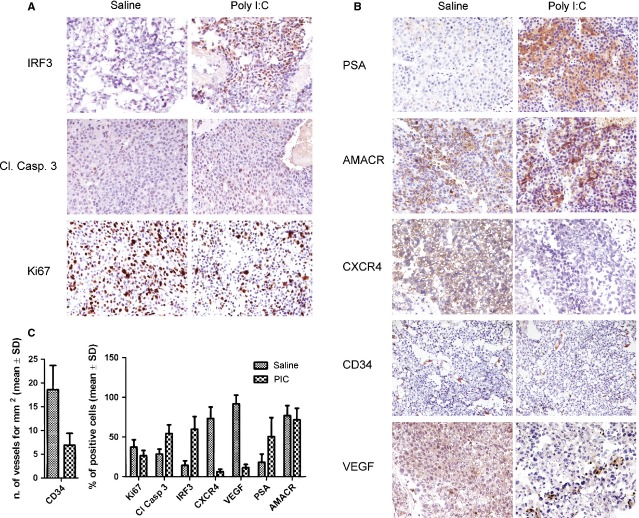
Immunohistochemistry of LNCaP xenografts. (A and B) 10 PIC treated (i.p.) and 10 saline treated tumours from two independent experiments were immunostained with the different antibodies. (C) Bar chart represents positive cells that were evaluated counting 10 high power fields (HPF). Values are reported as mean ± SD of the percentage of positive tumour cells of all the tumours. Mean number of vessels ± SD per mm^2^ tissue is shown.

The comparative histological analysis of poly I:C-treated and untreated LNCaP tumours showed that both intraperitoneal and intratumoural poly I:C administration affected the tumour features in a comparable fashion. In detail, poly I:C-treated tumour cells appeared unorganized, poorly cohesive and with large areas of necrosis, figures usually related to a regressive context. To further examine whether LNCaP tumours underwent cell cycle arrest and/or cell death following treatment with Poly I:C, the expression of Ki-67, a marker of proliferation and of the apoptotic marker cleaved caspase-3 were assessed in the xenograft tumours. When compared to vehicle alone, poly I:C treatment reduced the expression of Ki-67 and increased cleaved caspase 3 staining (Fig.7A and C), demonstrating the anti-proliferative and pro-apoptotic effects of poly I:C *in vivo*. Moreover, in accordance with our *in vitro* results, tumours treated with poly I:C showed nuclear localization of IRF-3, confirming the activation of this transcription factor in LNCaP xenografts (Fig.7A and C). Immunohistochemical analysis further showed that poly I:C treatment significantly increased tumour cell PSA expression, while not affecting AMACR and almost wiping out CXCR4 staining from cancer cells (Fig.7B and C). Finally, the analysis of angiogenesis markers showed that poly I:C treatment was followed by a change in vessel morphology, loss of well-structured walls and flimsy appearance and by a massive reduction in the density of CD34 positive tumour vessels (7 ± 3 *versus* 19 ± 5 vessels/mm^2^ of tumour surface, *P* < 0.0001; Fig.7B and C). Consistent with this data was the concomitant reduction of VEGF staining in poly I:C-treated cancer cells (Fig.7B and C). These findings point towards a central role of poly I:C in the survival and organization of endothelial cells *in vivo*.

### Expression of TLR3 and IRF-3 in human PCas

**Fig 8:**
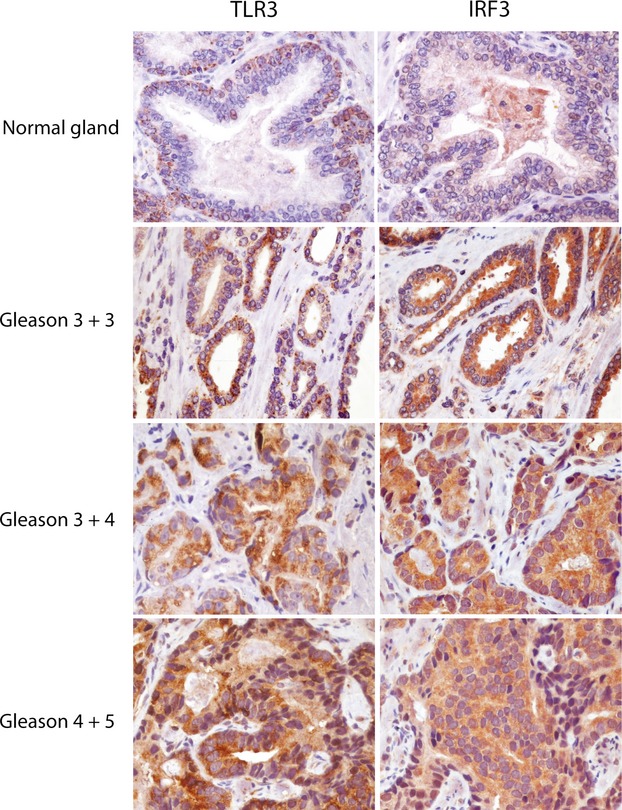
Expression of TLR3 and IRF-3 in normal prostate and prostate cancers. Human prostate biopsies were immune-stained as indicated. In normal glands, basal cells layer cytoplasm staining of TLR3 and diffuse faint granular cytoplasm distribution of IRF-3 were detected. Immunostaining of TLR3 and IRF-3 in increasing Gleason score PCa are shown (original magnification 400×).

To investigate TLR3 expression in human PCa and its association with IRF-3 expression, sections from 30 human prostate samples, harbouring cancers of different grade (10 tumours G1 = Gleason score ≤6; 10 tumours G2 = Gleason score 7; 10 tumours G3 = Gleason score ≥8) were immunostained with anti-TLR3 and anti-IRF-3 antibodies. We found that both proteins were expressed in normal prostate epithelium with a distribution, prevalent or limited, to the basal cell layer (Fig.8).

As reported in Table1, 26 out of 30 tumours tested expressed TLR3 while only one out of 30 different tumours was found negative for IRF-3. TLR3- and IRF-3- positive cell number and staining intensity was variable within the tumours but generally higher in PCa than in normal prostate gland (namely 68% *versus* 43% TLR3-positive cells and 83% *versus* 46% IRF-3-positive cells); moreover, the percentage of positive cells was found to significantly increase with the worsening of the tumour grade. As expected, TLR3 staining was cytoplasmatic and granular, while anti-IRF-3 stained diffusely the cytoplasm (Fig.8).

**Table 1:** TLR3 and IRF-3 expression in human prostate adenocarcinomas

	No. of positive cases*	% positive cells of G1 adenocarcinomas†	% positive cells of G2 adenocarcinomas†	% positive cells of G3 adenocarcinomas†
TLR3 cytoplasm staining	26/30	48 (23–100)	79 (28–100)	83 (22–100)
IRF-3 cytoplasm staining	29/30	72 (35–100)	86 (43–100)	95 (45–100)

* 10 cases for every grade where immunostained with TLR3 and IRF-3 antibodies by using conventional immunohistochemistry: G1 (4 tumours = Gleason 3 + 2; 6 tumours = Gleason 3 + 3), G2 (10 tumours = Gleason 3 + 4); G3 (4 tumours = Gleason 4 + 4; 3 tumours = Gleason 4 + 5; 3 tumours = Gleason 5 + 5).

† Results are reported as percentage mean (range) of positive tumour cells.

## Discussion

In this study, we demonstrate that the transcription factor IRF-3 plays an essential role in regulating TLR3-mediated apoptosis in LNCaP cells mainly through the activation of both intrinsic and extrinsic apoptotic pathways. Moreover, we show for the first time the direct anticancer effect of the synthetic TLR3 ligand poly I:C as a single therapeutic agent in a well-established human PCa xenograft model.

In infected cells viral dsRNA triggers the activation of specific transcription factors, such as IRF-3, a critical player in the induction of type I IFNs which inhibits virus replication (for a review see 29). Accordingly, we found that the stimulation of LNCaP cells with poly I:C induces the migration of IRF-3 into the nucleus and its phosphorylation, known to be necessary for the transcriptional activity of this protein. Moreover, both TRIF inhibitory peptide and bafilomycin completely impaired poly I:C-induced IRF-3 phosphorylation demonstrating the leading role of TLR3 in this process and excluding the involvement of other dsRNA cytosolic receptors. These data confirm that TLR3 is the main player in LNCaP response to poly I:C treatment, as previously shown by our and other groups 15,30. In addition to gene induction, virus infection can also trigger apoptosis and it has been recently reported that apoptotic pathway activated by paramyxovirus infection is IRF-3-dependent 31. The same IRF-3-dependent mechanism of poly I:C-induced apoptosis is effective also in human fibrosarcoma cells 27. Accordingly, here we demonstrate that the inhibition of IRF-3 phosphorylation or expression, through BX-795 or specific siRNA respectively, significantly impairs TLR3-mediated apoptosis, highlighting a crucial role of IRF-3 in the regulation of apoptosis in LNCaP cells. However, previous data have stated that IRF3-dependent apoptotic pathway in both virus infected and cancer cells is triggered only by the activation of dsRNA-sensing cytoplasmic receptors and not by endosomal receptor TLR3. To our knowledge, the present study demonstrates for the first time that IRF-3 is crucial for the TLR3-mediated induction of the intrinsic and extrinsic apoptotic pathway in LNCaP cells *in vitro*. It is known that IRF-3 can induce apoptosis through at least two different mechanisms. The first mechanism involves IRF-3-mediated transcription of the pro-apoptotic BH3 protein Noxa 32. The second mechanism occurs through the direct interaction of IRF-3 with the pro-apoptotic protein Bax, the consequent migration of this protein-complex to mitochondria and the resulting activation of the intrinsic apoptotic pathway 27,33. Nevertheless, our data suggest that even though IRF-3 regulates the transcription of Noxa, silencing of Noxa not only fails to protect LNCaP cells from poly I:C-induced apoptosis but surprisingly increases cell death, in line with Weber *et al*. data in melanoma cells 34.Further investigations are needed to better understand the molecular mechanism of IRF-3-mediated apoptosis in PCa cells.

As previously shown in breast and melanoma cancer cell lines 11,16,17, the pro-apoptotic effect of TLR3 signalling in PCa is restricted to some cell types 15. Here, we compared the effect of poly I:C in the normal prostate epithelial cells RWPE-1, tumourigenic androgen-sensitive LNCaP cells and androgen-insensitive LNCaP-derived C4-2B cells. The low sensitivity of RWPE-1 to poly I:C-induced apoptosis demonstrates that non-tumourigenic prostate epithelial cell viability is not markedly impaired by this treatment, although TLR3 stimulation activates the canonical signal transduction in this cell line (data not shown), therefore supporting the use of this compound *in vivo* as anticancer therapy. Accordingly, Besch *et al*. have previously shown that non-malignant epithelial cells are less sensitive than melanoma cells to poly I:C-induced apoptosis and our data support the hypothesis that TLR3-mediated apoptosis is selective for cancer cells also in the prostate 32,35. Among PCa cell lines tested for the apoptotic effect of poly I:C, the AR negative PC3 and Du-145 cells are resistant, whereas the AR positive LNCaP cells undergo a strong apoptosis. Therefore, to evaluate the role of AR in TLR3-mediated apoptosis we investigated the poly I:C effect in LNCaP cells treated with AR antagonist bicalutamide and also in a model of metastatic, castration resistant and AR positive PCa (LNCaP C4-2B cells). We observed that treatment of LNCaP cells with bicalutamide significantly reduced the apoptotic effect of poly I:C and that the androgen-insensitive LNCaP C4-2B were completely resistant to poly I:C-induced apoptosis, suggesting that the hormone sensitivity is involved in TLR3-mediated apoptosis. These data confirm our previous results obtained in PC3 cells overexpressing AR that resulted sensitive to poly I:C-induced apoptosis 15. Further experiments are needed to better clarify the mechanistic role of AR in TLR3-mediated apoptosis.

Chin *et al*. previously showed that anticancer activity of poly I:C in murine PCa cells (TRAMP-C2) subcutaneously engrafted in syngenic mice is mediated by immune cells, in particular NK cells 36. Conversely, Galli *et al*. demonstrated that poly I:C treatment reduced tumour growth only in combined therapy with retinoic acid in androgen-resistant human PCa in *in vivo* models 10. Our data demonstrate that poly I:C treatment, as a single therapeutic agent, markedly reduces the growth of LNCaP cells *in vivo* through a direct and therefore not immuno-mediated apoptotic effect on PCa cells. This new evidence highlights the possible application of poly I:C-based therapy for androgen-sensitive PCa, consistent with data obtained by Salaun *et al*. in breast cancer and melanoma. In fact, they demonstrated the anticancer efficacy of dsRNA treatment on human breast cancer and melanoma cells engrafted in immune-compromised mice. They also showed that treatment of breast cancer patients with dsRNA is associated with a reduction of metastatic relapse only in TLR3 positive tumours 11.

Our immunohistochemical study confirmed a reduction of the proliferative marker Ki-67 and an increase in cleaved caspase 3 staining in LNCaP xenografts treated with poly I:C. Moreover, poly I:C treatment significantly increased PSA expression in tumour cell and massively reduced anti-CXCR4 staining. The stromal derived factor-1 (SDF-1)/CXCR4 axis is associated with tumour aggressiveness and metastasis in PCa. Taken together these data suggest a possible role of poly I:C as differentiating agent. IHC also showed that poly I:C treatment reduced VEGF staining in LNCaP-derived tumours and angiogenesis *in vivo* supporting previous evidence showing that the activation of TLR3 signalling suppresses angiogenesis both *in vitro* and *in vivo* in other models 37,38.

Literature data have shown controversial results in studies of TLR3 expression in human normal prostate epithelium, benign prostatic hyperplasia and PCa. Gonzalez-Reyes *et al*. showed that 85% of the analysed PCa cases were positive for TLR3 expression and that high levels of TLR3 were associated with biochemical recurrence 39. On the other hand, it has been shown that TLR3 and IRF-3 were down-regulated in a subset of human PCa tissues and that TLR3-downregulation was associated with recurrence, suggesting a successful immune system escaping strategy for some advanced cancers 40. Salaun *et al*. have previously shown the predictive value of TLR3 expression in the response of breast cancer patients to dsRNA-based treatment 11. Our results show that TLR3 and IRF-3 are expressed in PCa tissues at higher level than in normal human prostate epithelium, supporting the potential application of poly I:C-based therapy, alone or in combination with standard anticancer treatments, for PCa.

## Supporting Information

**Figure S1.** TLR3 expression in LNCaP and RWPE-1 cells. Western blot of basal and poly I:C-stimulated TLR3 expression in LNCaP and RWPE-1 cells.

**Table S1.** Features of the antibodies used in immunohistochemical staining.
