# Supplementary figures and images for: TLR3 engagement induces IRF-3-dependent apoptosis in androgen-sensitive prostate cancer cells and inhibits tumour growth in vivo
Source: J Cell Mol Med. 2014 Dec 2;19(2):327–39. doi: 10.1111/jcmm.12379 (PMC4407608; doi:10.1111/jcmm.12379)

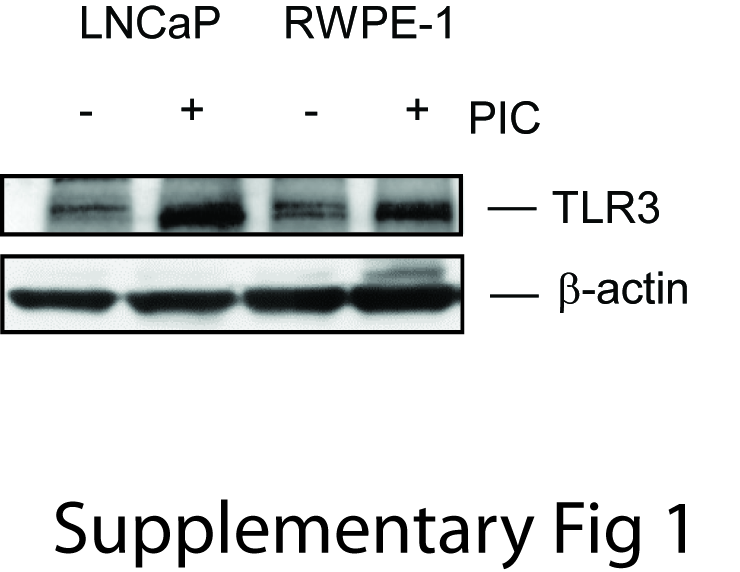

Supplement: Supplementary file 1 [file jcmm0019-0327-sd1.tif]
