# Supplementary material for: TLR3 engagement induces IRF-3-dependent apoptosis in androgen-sensitive prostate cancer cells and inhibits tumour growth in vivo
Source: J Cell Mol Med. 2014 Dec 2;19(2):327–39. doi: 10.1111/jcmm.12379 (PMC4407608; doi:10.1111/jcmm.12379)
Supplement: Supplementary file 2 [file jcmm0019-0327-sd2.doc]

| Antigen | Manufacturer | Clone | Isotype | Working dilution |
| --- | --- | --- | --- | --- |
| Human VEGFA | BD Pharmingen | G153-694 | Ms IgG2b k | 2.5 μg/ml |
| Human CXCR4 | BD Pharmingen | 12G5 | MsIgG2a K | 5 μg/ml |
| Mouse CD34 | BD Pharmingen | RAM34 | Rat IgG2 k | 2.5 μg/ml |
| Human IRF3 | Santa Cruz | FL-425 | Rab IgG pAb | 2 μg/ml |
| Human active Caspase-3 | Cell Signaling | (Asp175) (D3E9) | Rab IgG mAb | 1:250 |
| HumanTLR3 | Cell Signaling | D10F10 | Rab IgG mAb | 1:200 |
|  |  |  |  |  |
|  |  |  |  |  |
| Human PSA | Dako |  | Rab polyclonal | Ready to use |
| Human AMACR | Dako | 13H4 | Rab mAb | Ready to use |
| Human KI67 | Dako | MIB1 | Ms IgG1k | 1:150 |
| ISOTYPE CONTROL |  |  |  |  |
|  | BD Pharmingen | MCP1 | Ms IgG2b k | 5 μg/ml |
|  | Bd Pharmingen | G155-178 | Ms IgG2a K | 5 μg/ml |
|  | Bd Pharmingen | R35-95 | Rat IgG2a k | 2.5 μg/ml |
|  | Jakson Laboratories |  | Goat ant rabbit IgG | 2.5 μg/ml |

Supplementary Table I
